# Kinesiopathology, a Framework for Progressive Nature of Neuromusculoskeletal Dysfunctions: Implications for Physical Therapy

**DOI:** 10.1298/ptr.R0042

**Published:** 2026-06-30

**Authors:** Maiko MOROTANI

**Affiliations:** Nexus Motion, USA

**Keywords:** Kinesiopathology, Movement system, Neuromuscular activation pattern, Neuromusculoskeletal dysfunction

## Abstract

Kinesiopathology describes how suboptimal movement patterns generate progressive mechanical stress on tissues, contributing to structural changes and the onset of neuromusculoskeletal pain conditions. The purpose of this article is to discuss kinesiopathology as a framework for understanding the progressive nature of neuromusculoskeletal dysfunctions and its implications for physical therapy practice. Empirical evidence from biomechanical and clinical research across multiple conditions supports the relationship between altered movement patterns and musculoskeletal pathology. The nervous system consolidates frequently repeated movement patterns into automatized motor programs that operate below conscious awareness, perpetuating suboptimal tissue loading over time. This supports the reframing of traditionally defined orthopedic conditions as neuromusculoskeletal conditions, in which neuromuscular activation patterns govern movement behavior and influence tissue stress. Five phases of kinesiopathology are proposed, describing the progression from subtle, asymptomatic movement patterns through repetitive tissue loading, structural changes, and symptom development to chronic neuromusculoskeletal dysfunction. This framework highlights the clinical value of the early identification and modification of suboptimal movement patterns. Systematic movement system examination—including observation of alignment, neuromuscular activation patterns, and movement strategies during functional tasks—positions physical therapists to identify and address the movement-related mechanisms underlying neuromusculoskeletal conditions. How a person moves is not incidental to their neuromusculoskeletal health, but is fundamental to it. As movement system experts, the recognition and modification of movement dysfunction in its earliest phases represent both the defining competency and the greatest clinical opportunity of the profession.

## Introduction

Musculoskeletal dysfunctions represent one of the leading causes of pain, disability, and reduced quality of life worldwide^1)^. These conditions are particularly prevalent in aging populations and are associated with a substantial societal and economic burden^2,3)^. Japan is currently one of the world's most super-aged societies, with adults aged 65 years and older comprising more than one-quarter of the population and projected to approach 40% in the coming decades^4)^. As the population ages, the prevalence of chronic musculoskeletal conditions, such as low back pain, shoulder pain, and knee osteoarthritis^5)^, continues to rise. Among Japanese adults seeking care for chronic musculoskeletal pain—most commonly affecting the low back, neck, shoulder, and knee—approximately 30% reported no improvement or worsening of symptoms and expressed poor satisfaction with treatment despite receiving interventions^6)^. These conditions frequently persist for extended periods and are major contributors to functional limitations, reduced mobility, and decreased independence.

Many of the musculoskeletal pain conditions encountered in clinical practice cannot be fully explained by structural pathology alone. For example, imaging findings often show poor correlation with symptom severity, and individuals with similar structural findings may present with varied clinical manifestations^7)^. These observations suggest that additional factors beyond structural abnormalities contribute to the development and persistence of musculoskeletal pain. Therefore, attention has been increasingly directed toward the concept of kinesiopathology, a framework that describes the relationship between movement patterns and musculoskeletal conditions (Fig. 1)^8–10)^. Within this framework, the progression of subtle, suboptimal movements places excessive stress on specific structures, resulting in tissue injury, structural changes, and symptom development. Evidence from biomechanical and clinical research supports the relationship between altered movement patterns and various musculoskeletal pain conditions. For example, altered hip and knee mechanics have been associated with patellofemoral pain^11)^, while changes in scapular kinematics have been implicated in shoulder pathology^12)^.

**Fig. 1.**
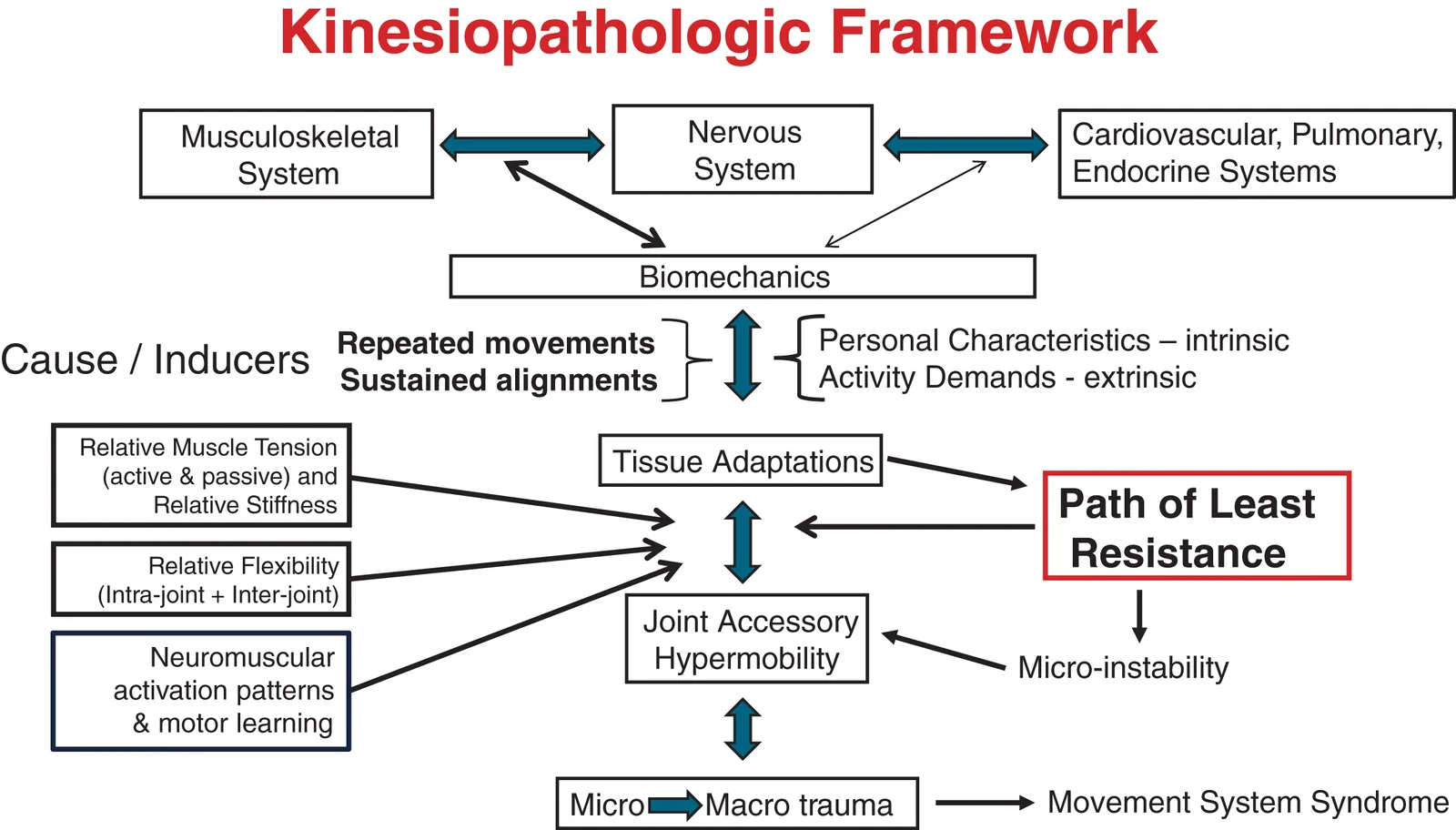
Kinesiopathologic framework.

The purpose of this article is to discuss the concept of kinesiopathology as a framework for understanding the progressive nature of musculoskeletal pain conditions. A conceptual model describing the progressive development of musculoskeletal pain conditions is proposed to facilitate movement-based clinical reasoning and to guide future research.

## The Movement System and Physical Therapy Identity

The analysis and optimization of human movement have long been central to the practice of physical therapy. However, the professional identity of physical therapists has historically been described in terms of the treatments performed rather than the body of knowledge underlying clinical decision-making. In many contexts, physical therapy has been defined primarily by interventions such as therapeutic exercise, manual therapy, and physical modalities. While these interventions are important components of care, defining the profession solely by the techniques performed obscures the underlying expertise required to evaluate and manage movement dysfunction.

In recognition of this concern, the American Physical Therapy Association formally adopted the concept of the movement system as a foundational identity for physical therapy practice, education, and research^13)^. Physical therapists possess unique training in evaluating the interaction among the muscular, skeletal, nervous, cardiovascular, pulmonary, endocrine, and integumentary systems and in identifying impairments that contribute to movement dysfunction^14)^. By emphasizing expertise in the movement system, the profession seeks to clarify its role in optimizing movement to improve health and functional outcomes across the lifespan^15)^.

## Empirical Evidence Linking Movement Patterns to Musculoskeletal Dysfunctions

Movement system diagnosis links clinical observations of movement to underlying biomechanical and physiological processes. For example, many studies examining anterior cruciate ligament (ACL) injury risk support the idea of kinesiopathology, highlighting that uncorrected hip adduction and femoral medial rotation are both risk factors for ACL injury and persistent deficit post-ACL reconstruction^16–18)^. In the 2025 systematic review, 83% of hip biomechanics studies showed increased ACL loading or an increased risk of ACL injury with medial knee alignment^19)^. The evidence demonstrates that as symptoms occur at the knee joint, the underlying contributing factors can be explained by suboptimal movement at the proximal segments.

The shoulder complex demonstrates how altered movement patterns contribute to mechanical stress on local tissues. During arm elevation, the scapula must upwardly rotate, posteriorly tilt, and externally rotate to maintain adequate space between the rotator cuff insertions and the surrounding bony and ligamentous structures. Individuals with shoulder impingement symptoms exhibit decreased scapular upward rotation, increased anterior tilt, and increased scapular internal rotation during humeral elevation compared to asymptomatic individuals, suggesting that altered scapular kinematics reduce the available subacromial space^20)^. Ludewig and Reynolds subsequently synthesized evidence across multiple shoulder pathologies—including rotator cuff tendinopathy, rotator cuff tears, glenohumeral instability, and adhesive capsulitis—confirming that scapular kinematic alterations are consistently associated with shoulder dysfunction^21)^. Furthermore, changes in glenohumeral and scapulothoracic kinematics are directly associated with changes in subacromial proximity, with the rotator cuff insertion found to be closest to the coracoacromial arch between 40° and 75° of humerothoracic elevation^22)^. Most recently, simulation studies have confirmed that reductions in scapular upward rotation of as little as 5° decrease the clearance between the rotator cuff tendon insertions and the glenoid at 120° of arm elevation, increasing the risk of internal impingement^23)^. Supraspinatus-to-glenoid contact occurs during standardized overhead reaching tasks, providing in vivo evidence that suboptimal scapular positioning during functional movement produces direct mechanical contact between soft tissue structures^24)^. Collectively, this line of research illustrates how habitual and suboptimal movement patterns progressively increase mechanical stress on rotator cuff tissues during repetitive overhead movements encountered in daily activities and sport.

Chronic low back pain provides further evidence of the relationship between habitual movement patterns and symptom provocation. People with low back pain demonstrate earlier and greater lumbopelvic rotation during prone knee flexion and prone hip lateral rotation than back healthy group^25)^. Subjects with low back pain exhibit greater early lumbar excursion during functional movements such as picking up an object^26)^. The amount of early-phase lumbar excursion was significantly associated with the amount of change in pain^27)^. Person-specific motor skill training outperformed a generic strengthening and stretching exercise program at 3 weeks and 6 weeks of treatment, with improvements maintained at 6 and 12 months post-treatment^28)^. The motor skill training was based on a systematic examination used to classify subjects into movement system diagnosis.

The commonality across this evidence is not necessarily the “what” but the “how” of movement patterns. Despite the recognition that problematic movement cannot be explained by range of motion, manual muscle testing, isolated impairments, and special tests, these measures continue to be the predominant clinical assessments performed by physical therapists. Examination of the quality of movement and its effects on tissue stress would be the most relevant clinical assessment. By integrating principles from biomechanics, motor learning, and tissue adaptation, the kinesiopathologic framework guides clinical reasoning toward how movement patterns influence mechanical loading. Thus, movement-based interventions address symptom management as well as prevention and progression of musculoskeletal dysfunctions.

## Neuromuscular Activation Patterns and their Influence on Movement

Musculoskeletal pain conditions frequently develop gradually over time rather than resulting from a single acute injury. Many individuals report a progressive onset of symptoms associated with routine daily activities such as walking, stair negotiation, reaching, prolonged sitting, sleeping, or performing sports-related skills. One important factor influencing movement behavior is the interaction between the nervous and muscular systems in accomplishing functional tasks. The central nervous system often has movement solutions that minimize effort or decrease energy demands^29)^. Such optimization of neuromuscular strategies allows individuals to complete tasks efficiently^30)^; however, the most energetically efficient movement pattern is not usually the one that minimizes stress on specific tissues. As a result, individuals repeatedly perform tasks using movement strategies that are energy-efficient and automatic yet biomechanically stressful to specific joints and structures. Over time, the repeated use of these strategies contributes to the accumulation of mechanical stress within the musculoskeletal system^31)^. Since the nervous system’s adaptations are intertwined in how individuals move, it may be more appropriate to reframe what are traditionally considered orthopedic conditions as neuromusculoskeletal conditions. While the term “musculoskeletal” captures the structural tissues involved, it does not fully reflect how neuromuscular activation patterns govern movement behavior and therefore influence the mechanical stress placed upon tissues. Treatment that addresses only the structural or symptomatic components of a condition and not the underlying neuromuscular activation patterns is missing the intervention of the primary problem. Recognizing the neuromusculoskeletal nature of these conditions shifts the focus from the treatment of isolated impairments to the movement pattern itself, which is the key problem.

## Progressive Nature of Neuromusculoskeletal Dysfunctions

Biological tissues can adapt to mechanical stress; however, when the load exceeds the capacity of the tissue to adapt, structural changes occur^32)^. For example, increased mechanical loading of the medial compartment of the knee, often estimated using the knee adduction moment during gait, is associated with the progression of medial tibiofemoral osteoarthritis^33)^. Similarly, habitual forward head posture progressively increases compressive loading on the cervical intervertebral discs and facet joints. Biomechanical evidence demonstrates that cervical sagittal malalignment—a postural consequence particularly prevalent in aging populations with increased thoracic kyphosis—produces pathomechanical changes, including altered disc and facet loading, compensatory segmental hyperextension, and changes in neural foraminal dimensions^34)^. These findings support the notion that repeated mechanical loading patterns influence structural changes over time.

The kinesiopathologic framework includes multiple key components, such as relative joint flexibility, relative muscle stiffness, motor learning principles, path of least resistance, and development of microinstability^8,9)^. The progression from subtle alignment and movement deviations to symptomatic pathology can be described in progressive phases. The proposed progressive phases of kinesiopathology describe how movement patterns influenced by automatic neuromuscular activation pattern contribute to the development of neuromusculoskeletal pain conditions (Table 1).

**Table 1.** Proposed phases of kinesiopathology

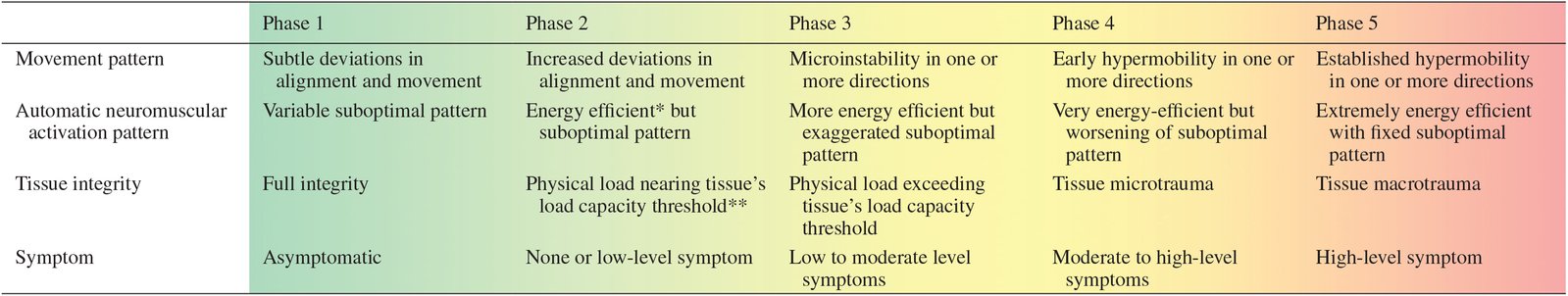

*Energy efficiency does not equate to optimal biomechanical condition. When a suboptimal pattern becomes energetically efficient, the nervous system reinforces it as the preferred motor strategy. This perpetuates the very movement pattern that excessively loads tissues, creating a self-reinforcing cycle that is difficult to interrupt without deliberate intervention.

**Load capacity threshold: threshold for adaptation such that, when exceeded, the tissue enters the injury zone

Phase 1 involves subtle deviations in alignment or movement that are often asymptomatic. These deviations include altered joint alignment, changes in segmental coordination, or movement strategies that are adopted during functional activities. Because these patterns do not initially produce symptoms, they persist unnoticed for extended periods of time. The origins of Phase 1 subtle deviations are multifactorial and include habitual posture, occupational demands, sport-specific motor learning, and movement strategies acquired during formative periods of physical development. A central motor learning principle relevant to the progression of neuromuscular activation pattern (second row, Table 1) is that the nervous system consolidates frequently repeated movement patterns into highly automatized motor programs that eventually operate below the level of conscious awareness^35,36)^. Over time, these learned strategies generalize beyond their original context and begin to appear across a broad range of functional tasks. A clinical example is the ballet-trained individual who, when asked to perform a straight leg raise in supine, automatically points the foot. This is a movement strategy deeply ingrained through years of repetitive practice, yet one that is now expressed during a routine clinical test entirely unrelated to ballet. This example demonstrates how sport-specific or occupation-specific motor learning produces movement patterns that persist long after the original training context has ended. In Phase 1, neuromuscular activation pattern is more variable and suboptimal; however, as repeated activity exposure continues, it becomes progressively less variable and more energy-efficient. This perpetuates the suboptimal pattern that becomes self-reinforcing.

With continued daily activities, these movement patterns result in repetitive mechanical loading of specific tissues (Phase 2; Table 1). Repeated loading associated with routine tasks such as walking^37)^, lifting^38)^, or reaching^39)^ gradually increases stress on structures, with the physical load nearing the tissue’s load capacity threshold. Despite the tissue’s capacity to adapt to mechanical stress, prolonged suboptimal loading challenges the tissue’s structural integrity.

When cumulative mechanical stress exceeds the tissue’s load capacity threshold, structural or physiological changes begin to occur (Phase 3; Table 1). During Phase 3, the movement pattern progresses to microinstability of the involved joint in one or more directions, and the suboptimal neuromuscular activation pattern becomes increasingly energy-efficient. Low- to moderate-level symptoms are present during specific movements or tasks that increase tissues’ mechanical stress. In the knee joint, for example, altered gait mechanics and increased medial compartment loading have been associated with the structural progression of osteoarthritis^40)^, illustrating how repeated deviations in loading patterns contribute to alterations in tissue integrity over time.

As structural changes progress to tissue microtrauma, individuals begin to experience moderate- to high-level symptoms (Phase 4; Table 1). Movement pattern deviations progress to early hypermobility in one or more directions, and the neuromuscular activation pattern continues to worsen—becoming highly energy-efficient but increasingly maladaptive. When the underlying movement behaviors and symptoms remain unresolved, the condition eventually transitions to a chronic state (Phase 5; Table 1). At this phase, established hypermobility and a fixed suboptimal neuromuscular activation pattern coexist with ongoing mechanical stress and changes in pain-processing mechanisms, including increased sensitivity within the nervous system. This progression from subtle movement deviations to chronic neuromusculoskeletal dysfunction is why early identification of suboptimal movement patterns related to automatic neuromuscular activation patterns, tissue integrity, and symptoms represents a clinically meaningful opportunity for intervention.

### A Wake-up Call: The Importance of Movement and Movement Expertise

In his 57th McMillan Visionary Leadership Lecture, titled “Movement Matters: The Path to Professional Impact,” Powers charged physical therapists to “fully embrace our identity as movement experts and consistently embody it.”^41)^ Just as radiologists are expected to provide their expertise in interpreting magnetic resonance imaging (MRI) or X-ray imaging, physical therapists should be expected to deliver the movement expertise in the analysis of patient’s movements. A deep and systematic understanding of optimal and suboptimal movement patterns, as well as the underlying mechanisms that drive them, is what empowers physical therapists to select appropriate therapeutic exercises, guide functional task training, and determine effective movement interventions that address the root causes rather than its symptomatic manifestations. In the same McMillan Lecture, Powers stated, “When the underlying movement problem remains unaddressed, pain becomes recurrent, persistent, and eventually chronic.” ^41)^ Physical therapists can intervene not only after symptoms begin to impact patients’ lives (Phases 3–5; Table 1) but also before tissues undergo changes in the earlier phases (Phases 1 and 2; Table 1).

### Limitations of Current Evidence and Clinical Implementation

Although growing evidence supports the relationship between movement patterns, mechanical loading, and musculoskeletal dysfunctions, several limitations remain in aspects of kinesiopathology. Individual tissue tolerance is highly variable and is influenced by activity. Some individuals may remain in Phases 1 and 2 for a longer period despite the persistence of suboptimal patterns, while others with lower load capacity threshold may progress more quickly to Phases 3–5 (Table 1).

Although biomechanical studies have demonstrated associations between altered movement mechanics and conditions such as ACL tear, patellofemoral pain, rotator cuff tendinopathy, low back pain, and knee osteoarthritis, the complex interactions between mechanical loading, tissue adaptation, and symptom development remain to be understood. Longitudinal studies examining the progression from early movement deviations to structural tissue changes and symptomatic conditions are still relatively limited. More studies that examine changes in symptom level with changes in movement pattern will also benefit educators and clinicians.

In addition, neuromusculoskeletal dysfunctions are multifactorial in nature. Biological, psychological, and social factors all contribute to the experience of pain and disability. The kinesiopathologic framework emphasizes the role of mechanical loading and movement behavior. However, this perspective should be considered within the broader context of contemporary models of pain that recognize the interaction between biomechanical and neurophysiological mechanisms^42)^. Further research is needed to clarify how movement-related mechanical factors interact with other contributors to neuromusculoskeletal pain, including central sensitization and psychosocial influences.

Another limitation relates to the translation of the kinesiopathologic framework into routine clinical practice. In many clinical environments, time constraints and productivity demands limit the extent to which detailed movement analysis can be performed.

Educational models within physical therapy programs also influence how movement system concepts are applied in practice. In some physical therapy educational curricula, content related to various physiological systems is taught in separate courses, which may unintentionally reinforce a fragmented approach to patient evaluation^43)^. This structure makes it more challenging for students to develop an integrated understanding of how multiple systems interact to produce optimal or suboptimal movement. Additionally, how new graduates practice is greatly influenced by their clinical education. Knowledge gained in classrooms or in clinical settings is amplified when students and practitioners can translate it into applied practice. In both educational programs and clinical practice, greater emphasis on movement analysis, task-specific assessment, and clinical reasoning related to movement dysfunction helps strengthen the integration of movement system knowledge within the profession.

## Implications for Clinical Practice: The Role of Physical Therapists as Movement System Experts

Despite these limitations, the kinesiopathologic framework provides a clinically meaningful structure for understanding how movement patterns contribute to the development and progression of neuromusculoskeletal conditions. Future research priorities should include longitudinal studies examining progression from early movement deviations to structural tissue changes, clinical trials evaluating the effectiveness of movement-based interventions across specific neuromusculoskeletal conditions, and studies directly examining how changes in neuromuscular activation patterns correlate with changes in tissue loading and symptom levels. Such evidence will be essential for refining the proposed progressive phases and establishing the kinesiopathologic framework as an evidence-based guide for clinical practice.

Systematic examination of the movement system includes observation of alignment, simple joint movements performed actively and passively, neuromuscular activation patterns, and movement strategies during task-specific activities. This information allows clinicians to identify the movement behaviors that contribute to tissue stress and symptom provocation. The progressive phases described in this article highlight the value of early identification and modification of suboptimal movement patterns before the progression of symptoms. How a person moves is not incidental to their neuromusculoskeletal health but is fundamental to it. As movement system experts, recognizing and addressing movement dysfunction at its earliest phases represent both the defining competency and the greatest clinical opportunity for physical therapists.
